# RhNRG-1β Protects the Myocardium against Irradiation-Induced Damage via the ErbB2-ERK-SIRT1 Signaling Pathway

**DOI:** 10.1371/journal.pone.0137337

**Published:** 2015-09-02

**Authors:** Anxin Gu, Yamin Jie, Liang Sun, Shuping Zhao, Mingyan E, Qingshan You

**Affiliations:** 1 Department of Radiotherapy, the Affiliated Tumor Hospital of Harbin Medical University, Harbin, Heilongjiang, China; 2 Department of Radiotherapy, the Fourth Affiliated Hospital of Harbin Medical University, Harbin, Heilongjiang, China; 3 Department of Human Anatomy, Harbin Medical University, Harbin, Heilongjiang, China; University of Cincinnati, College of Medicine, UNITED STATES

## Abstract

Radiation-induced heart disease (RIHD), which is a serious side effect of the radiotherapy applied for various tumors due to the inevitable irradiation of the heart, cannot be treated effectively using current clinical therapies. Here, we demonstrated that rhNRG-1β, an epidermal growth factor (EGF)-like protein, protects myocardium tissue against irradiation-induced damage and preserves cardiac function. rhNRG-1β effectively ameliorated irradiation-induced myocardial nuclear damage in both cultured adult rat-derived cardiomyocytes and rat myocardium tissue via NRG/ErbB2 signaling. By activating ErbB2, rhNRG-1β maintained mitochondrial integrity, ATP production, respiratory chain function and the Krebs cycle status in irradiated cardiomyocytes. Moreover, the protection of irradiated cardiomyocytes and myocardium tissue by rhNRG-1β was at least partly mediated by the activation of the ErbB2-ERK-SIRT1 signaling pathway. Long-term observations further showed that rhNRG-1β administered in the peri-irradiation period exerts continuous protective effects on cardiac pump function, the myocardial energy metabolism, cardiomyocyte volume and interstitial fibrosis in the rats receiving radiation via NRG/ErbB2 signaling. Our findings indicate that rhNRG-1β can protect the myocardium against irradiation-induced damage and preserve cardiac function via the ErbB2-ERK-SIRT1 signaling pathway.

## Introduction

Radiation-induced heart disease (RIHD) is one of the important long-term side effects of radiotherapy for thoracic tumors, breast cancer, chest wall tumors and lymphoma when all or part of the heart is exposed to ionizing radiation [1, 2]. The resultant conditions can include pericardial and atherosclerosis, myocardial infarction, cardiac valve injuries, myocardial fibrosis, and conduction abnormalities. Numerous studies addressing RIHD suggest that radiation causes micro- and macro-vascular endothelial inflammatory responses, which can trigger vascular damage and myocardial degeneration [1, 3]. In addition, many studies indicate that the endocardium and endothelial cells of the myocardial microvasculature regulate the proliferation, maturation, injury and regeneration of the myocardium through multiple paracrine signaling pathways [4]. However, few studies focus on the cross-talk between the endocardium and myocardium in RIHD.

Neuregulin (NRG)-1/ErbB2 signaling is one of the important pathways that mediates the cross-talk between the endocardium and myocardium [5, 6]. NRG-1 is a member of the neuregulin family that is synthesized and expressed in the endocardium and the endothelial cells of the myocardial microvasculature. NRG-1 gene encodes more than 14 soluble and transmenbrane protein products [5]. In mammalian myocardium tissue, soluble NRG-1 isoforms (mainly NRG-1β) released from the proteolysis of the extracellular domain of transmembrane NRG-1 isoforms function as a paracrine signaling molecule that binds the tyrosine kinase receptor ErbB4 and activates ErbB4 and its coreceptor ErbB2 co-expressed on adjacent cardiomyocytes [6, 7]. Although unable to directly bind ligands including NRG-1β, ErbB2 serving as the preferred heterodimerization partner for ErbB4 is essential for the stabilization of ligand binding and ligand-induced receptor signaling transduction [8, 9]. When activated by NRG-1, ErbB2 signaling promotes the survival and proliferation of cardiomyocyte, preserves the myocardial sarcomeric structure, maintains intracellular Ca^2+^ homeostasis and enhances cardiac pump function, which is considered to be a prospective target in the treatment of heart damage [10, 11].

Based on these findings, a 61-amino acid recombinant human neuregulin-1β protein (rhNRG-1β), which is encoded by the *NRG* gene and contains an immunoglobulin domain and the EGF-like domain necessary for ErbB2/ErbB4 activation, has been applied in preclinical and clinical studies of heart disease treatment. In preclinical studies, rhNRG-1β improved cardiomyocyte survival and cardiac function in models of dilated cardiomyopathy and ischemic, viral and doxorubicin-induced heart damage [7, 9, 12–14]. Recently, rhNRG-1β was demonstrated to improve the cardiac function of patients with chronic heart failure in a phase II clinical trial [15]. However, the effects of rhNRG-1β on RHID have not been reported to date. In this study, we investigated the impacts of rhNRG-1β on the irradiated myocardium of rats along with related mechanisms, aiming to develop a new avenue for RHID treatment.

## Materials and Methods

### Ethics Statement

All procedures involving animals were performed strictly according to the guidelines of the Harbin Medical University Animal Care and Use Committee and approved by the Harbin Medical University Animal Care and Use Committee.

### Experimental animals

Male Sprague-Dawley rats weighing 200–250 g were obtained from Harbin Medical University Animal Center (Harbin, Heilongjiang, China) and maintained under a 12:12 light-dark cycle, with free access to food and water as described previously [16]. Rats were randomly divided into four groups (24 rats/group): the Control group, the Vehicle group, the NRG group and the H+NRG group. The Control group received neither irradiation nor drug administration. The rats in the NRG group received irradiation and intravenous injection of rhNRG-1β (15 μg/kg; PeproTech, USA) in vehicle (0.9% saline, pH 7.4) 3 days before and 7 days after irradiation, while the animals in the Vehicle group received only irradiation and injection of an equal amount of vehicle at the same time points. The rats in the H+NRG group were subjected not only to irradiation and administration of rhNRG-1β at the same dose as in the NRG group but also to intravenous injection of Herceptin (15 μg/kg; Roche, USA) 1 h before rhNRG-1β injection. For all groups, weight and grooming were analyzed weekly to monitor overall health and maintenance behavior. No unexpected death was observed throughout the observation. The rats were subjected to immunostaining for γ-H2AX and to Western blotting for γ-H2AX, cleaved caspase 3, SIRT1 and phosphorylated and total ERKs in myocardium tissue 6 h after irradiation, to assessment of cardiac function and myocardium ATP levels 1 week before and 1, 5, 10, 15 and 20 weeks after irradiation, and to measurement of the average cardiomyocyte area as well as myocardium fibrosis 20 weeks after irradiation. For cleaved caspase 3 analysis, Doxorubicin (20 mg/kg, *i*.*v*., 5d; Sigma-Aldrich, USA)-treated rats were used as positive control [17].

### Cardiomyocyte isolation and culture

Healthy male Sprague-Dawley adult rats (200–250 g) were anesthetized with 4% chloral hydrate and sacrificed by cervical dislocations. Fresh rat hearts were dissected, harvested and minced into 1–3 mm^3^ pieces in Hank’s Balanced Salt Solution (Invitrogen, USA), followed by incubation with collagenase B (1.8 mg/ml, Roche, USA) and D (2.4 mg/ml, Roche) in DMEM/F-12 (Invitrogen) at 37°C for 12 hours. These samples were subsequently pipetted repeatedly and filtered through a 200-mesh strainer. The supernatant was centrifuged (500 rpm for two minutes) to collect the isolated cardiomyocytes. Following differential attachment to eliminate fibroblasts, the cardiomyocytes were plated onto poly-L-lysine (Invitrogen)/laminin (Invitrogen)-coated 6-well plates or coverslips at a cell density of 1×10^5^/ml using a wide-bore pipette and maintained in DMEM/F-12 (Invitrogen) containing 10 μM cytosine 1-β-D-arabinofuranoside, 10% fetal calf serum (FCS) (Invitrogen), 100 mg/ml streptomycin, 100 IU/ml penicillin (Invitrogen) and 20 mM creatine monohydrate (Sigma-Aldrich) for 48 h. The cultured cardiomyocytes were allocated to five groups: the Control group, the Vehicle group, the NRG group, the H+NRG group, M+NRG group and the F+NRG group. The cells in the control group received neither irradiation nor drug treatment. The cells in the NRG group were supplemented with 1 μg/ml rhNRG-1β in vehicle (0.9% saline, pH 7.4) 3 d before irradiation, while the cells in the vehicle group received only irradiation and injection of an equal amount of vehicle at the same time point. The cells in the H+NRG group, M+NRG group and the F+NRG groups were subjected not only to irradiation and pre-irradiation treatment with rhNRG-1β at the same concentration as in the NRG group but also to supplementation with Herceptin (6.4 μg/ml; Roche), FR180204 (50 μM, Sigma-Aldrich) and Mubritinib (25 μM, Sigma-Aldrich), respectively, 1 h before rhNRG-1β treatment. Cells in the Control, Vehicle, NRG, H+NRG and M+NRG groups were subjected to immunostaining and Western blotting for γ-H2AX or cleaved caspase 3, in addition to measurements of the mitochondrial membrane potential (MMP), ATP levels and cytochrome *c* oxidase (CCO) and succinate dehydrogenase (SDH) activity and Western blotting for SIRT1, phosphorylated and total MAPKs six hours after irradiation. The cells in the F+NRG group underwent Western blotting for SIRT 1 six hours after irradiation. For cleaved caspase 3 analysis, Doxorubicin (2 μM, *i*.*v*., 12 h; Sigma-Aldrich)-treated cells were used as positive control [18].

### Cardiomyocyte irradiation and local heart irradiation in rats

Cardiomyocytes growing on 6-well plates were irradiated in a 15×15 cm^2^ square field with 5 Gy (6 MeV electron rays, 6A, dose rate of 4 Gy/min).

For local heart irradiation on rats, animals were anesthetized with 4% chloral hydrate and placed supinely on a plate with limbs and head fixed. They were subjected to CT scanning, and the heart was outlined for radiotherapy planning simulation using a treatment-planning system (Elekta PrecisePlan 2.16, Elekta AB, Stockholm, Sweden) to determine whether the 90% isodose curve included the heart. For irradiation, a low-melting lead alloy was attached to the medical linear accelerator treatment system (Elekta Synergy, Sweden) to generate a 2×2 cm^2^ square anterior-posterior radiation field, in which rat hearts were exposed to 21 Gy radiation (6 MeV electron-rays, 6A, dose rate of 6 Gy/min). Ibuprofen (20 mg/kg; Sigma-Aldrich) was injected *i*.*p*. to rats in all groups for 3 days after irradiation in order to suppress irradiation-induced pain.

### Immunostaining

For adult cardiomyocyte cultures or frozen sections of rat myocardium tissue, samples were washed with cold PBS (pH 7.4), fixed with 4% paraformaldehyde in PBS at room temperature for 10 min, permeabilized in 0.5% Triton X-100 for 5 min and blocked with PBS containing 3% bovine serum albumin (BSA) and 0.05% Triton X-100 for 1 h. Subsequently, the cells were incubated with a primary antibody against γ-H2AX (rabbit IgG; 1:300; Sigma-Aldrich, USA) in 1% BSA at 4°C overnight and then with Alexa Fluor 594-goat anti-rabbit IgG (1:1,000; Invitrogen, USA) at room temperature in the dark for 1 h. Nuclei were stained with 4',6-diamidino-2-phenylindole (DAPI; 1μg/ml; Roche) for 5 minutes, and the samples were analyzed under fluorescence microscope (BX51; Olympus, Japan) or confocal microscopy (IX83, Olympus, Japan). The fluorescence intensity of γ-H2AX in per random filed were measured and the average fluorescence intensity of γ-H2AX per cardiomyocyte were calculated using Image Pro Plus 6.0 software.

### ATP measurements

The ATP levels in cultured adult cardiomyocytes or rat myocardium tissue were measured in accordance with the manufacturer's instructions (ATP Colorimetric/Fluorometric Assay Kit, Sigma-Aldrich). To prepare samples for the assay, 1×10^6^ cells were lysed, or 10 mg of tissue was homogenized in 100 μL of ATP Assay Buffer and then deproteinized using a 10 kDa MWCO spin filter. To analyze ATP levels, the fluorescence (RLU, λ _excitation_ = 535/ λ _emission_ = 587 nm) of the appropriate reaction mixture was measured in a microplate reader (SpectraMax M2, Molecular Devices Corporation, USA).

### Isolation of mitochondria

Mitochondria of cardiomyocytes were isolated as reported in previous study [19, 20]. Briefly, cardiomyocytes were washed with cold PBS and homogenized with a Dounce glass homogenizer in H-medium (70 mM sucrose, 220 mM mannitol, 2.5 mM HEPES, pH 7.4, 2 mM EDTA containing 1 mM PMSF, 1 μg/ml each of pepstatin, leupeptin, aprotinin and antipain). Rat heart tissues were finely minced and disrupted in a polytron tissue homogenizer using two pulses of 5 seconds each. The crude extract was then homogenized in a Dounce glass homogenizer. Subcellular fractions were prepared through differential centrifugation. Nuclei and intact cells were pulled down via centrifugation at 1,000 x *g* for 10 min at 4°C. Then, the mitochondrial fraction was obtained through centrifugation of the supernatant at 10,000 x *g* for 10 min at 4°C. The purified mitochondria were subjected to measurement of the mitochondrial membrane potential (MMP) or were suspended in mitochondrial storage fluid (300 mmol/L sucrose, 2 mmol/L Hepes, 0.1 mmol/L EGTA, pH7.4) and stored at -80°C.

### Measurement of the mitochondrial membrane potential (MMP)

Purified mitochondria were incubated with rhodamine labeling solution (10 μg/ml rhodamine 123 in PBS) for 15 min and analyzed with a microplate reader (SpectraMax M2, Molecular Devices Corporation) to measure the fluorescence intensity at a wavelength of 534 nm, with an excitation wavelength of 504 nm. (for rhodamine 123, e_xcitation_ = 504 nm; e_mission_ = 534 nm.)

### Measurement of cytochrome *c* oxidase and succinate dehydrogenase activities

Cytochrome *c* oxidase (CCO) and succinate dehydrogenase (SDH) activities were measured using a CCO assay kit (Invitrogen) and an SDH assay kit (Invitrogen) according to manufacturer’s instructions as described in previous study [20]. Briefly, CCO activity was quantified as the change in the absorbance at 550 nm under a spectrophotometer, where the cardiac mitochondrial protein catalyzed reduced cytochrome *c* into oxidized cytochrome *c*. Additionally, SDH activity was further calculated according to the rate of the 2,6-DPIP reduction reaction, as when the cardiac mitochondrial protein catalyzes the substrates, FAD is reduced to FADH, coupled with a 2,6-DPIP reduction reaction.

### Western blotting

Western blotting was performed as described previously [21, 22]. Briefly, cell samples were lysed in a buffer containing 10 mM Tris (pH 7.4), 100 mM NaCl, 1 mM EDTA, 1 mM EGTA, 1 mM NaF, 20 mM Na_4_P_2_O_7_, 2 mM Na_3_VO_4_, 0.1% SDS, 0.5% sodium deoxcholate, 1% Triton-X 100, 10% glycerol, 1 mM PMSF, 60 mg/mL aprotinin, 10 mg/mL leupeptin, and 1 mg/mL pepstatin. After centrifugation at 12,000 x *g* for 10 min, the protein concentration was measured using the bicinchoninic acid (BCA) protein assay (Sigma-Aldrich) and 15 mg of protein was then loaded into an SDS-PAGE gel for separation. Western blotting was conducted using the Multiphor II Electrophoresis System (Bio-Rad Laboratories, Inc.) at 200 mA for 1.5 h. After blocking with 5% skim milk in TBST (0.1% Triton X-100 in TBS) for 1 h at room temperature, the blots were incubated with an anti-γ-H2AX (rabbit IgG; 1:1000; Sigma-Aldrich), anti-caspase 3 (rabbit IgG; 1:1,000; Proteintech Group, Inc., USA), anti-cleaved caspase 3 (rabbit IgG; 1:1,000; Proteintech Group, Inc.), anti-SIRT1 (rabbit IgG; 1:1,000; Proteintech Group, Inc.), anti-ERK1/2 (rabbit IgG; 1:1,000; Santa Cruz Biotechnology, Inc., USA), anti-p-ERK1/2 (rabbit IgG; 1:1,000; Santa Cruz Biotechnology, Inc.), anti-P38 (rabbit IgG; 1:1,000; Proteintech Group, Inc.), anti-p-P38 (rabbit IgG; 1:1,000; Proteintech Group, Inc.), anti-JNK (rabbit IgG; 1:1,000; Proteintech Group, Inc.), anti p-JNK (rabbit IgG; 1:1,000; Proteintech Group, Inc.) or anti-β-actin (rabbit IgG; 1:1,000; Cell Signaling Technology, USA) primary antibody overnight at 4°C. Following incubation with a horseradish peroxidase-conjugated secondary antibody (goat anti-rabbit IgG; 1:5,000; Invitrogen) for 1 h, the immunoreactive bands were detected using the enhanced chemiluminescence western blotting detection reagent (GE Healthcare Bio-Sciences) on film. Densitometric analysis was performed using Image Pro Plus 6.0 software.

### Assessment of cardiac function via echocardiography

Echocardiographic images of hearts from all groups of rats were obtained using a 12-MHz ultrasound probe and an echograph (SONOS 5500, Hewlett Packard) while rats were immobilized under anesthesia (3% isoflurane) as reported previously [23]. The parasternal short-axis view was employed to image the heart in two dimensions at the level of the papillary muscles. LV end-diastolic (LVEDV) and end-systolic volumes (LVESV) were measured using software from the ultrasonography and LV ejection fraction (EF) was calculated using the formula (LVEDV–LVESV)/ LVEDV×100%. LV fractional shortening (FS) was calculated from M-mode-derived left ventricular inner diameters in systole (LVIDs) and diastole (LVIDd) using the formula (LVIDd–LVIDs)/LVIDd×100%. All imaging and interpretation of results was carried out blinded to the treatment group.

### Histological assessment

Histological assays were conducted as described in previous studies [24, 25]. In brief, rats were successively perfused with 0.9% saline and 4% paraformaldehyde in PBS. Their hearts were dissected, post-fixed in 4% paraformaldehyde, dehydrated in 70%, 80%, 90%, 95% and 100% ethanol, vitrified with xylene, processed in paraffin with gradient melting points, embedded in paraffin (60–62°C) and sectioned on a microtome (Leica, Germany) into 4-μm coronal slices, then subjected to hematoxylin and eosin (HE) or Masson’s trichrome staining.

HE staining was performed according to the standard protocol. After sections were photographed under an optical microscope (Olympus, Japan), the average cardiomyocyte area was measured using Image Pro Plus 6.0 software.

For Masson’s trichrome staining, sections were deparaffinized and then embedded in a Masson composition solution and a light green silk fibroin solution to evaluate changes in interstitial fibrosis. The total collagen content and percentage of fibrotic areas in each section were assessed with Image Pro Plus 6.0 software.

### Data analysis

Data processing and analysis were conducted as reported previously [21, 24]. For all of the assessments, at least three independent experiments (n as detailed in the Results) were performed, and statistical processing was conducted using Microsoft Excel and GraphPad Prism 5.0 software. The data are expressed as the mean ± standard deviation (SD) or mean ± standard error of the mean (SEM). One-way or two-way analysis of variance (ANOVA) for repeated measures, followed by Bonferroni’s post hoc test for multiple comparisons, was used to determine statistically significant differences. P values<0.05 were considered statistically significant. All of the images acquired via optical or fluorescence microscopy were analyzed with Image Pro Plus 6.0 and processed using Adobe Photoshop.

## Results

### rhNRG-1β rescues radiation-induced myocardium injury via ErbB2 signaling

**Fig 1:**
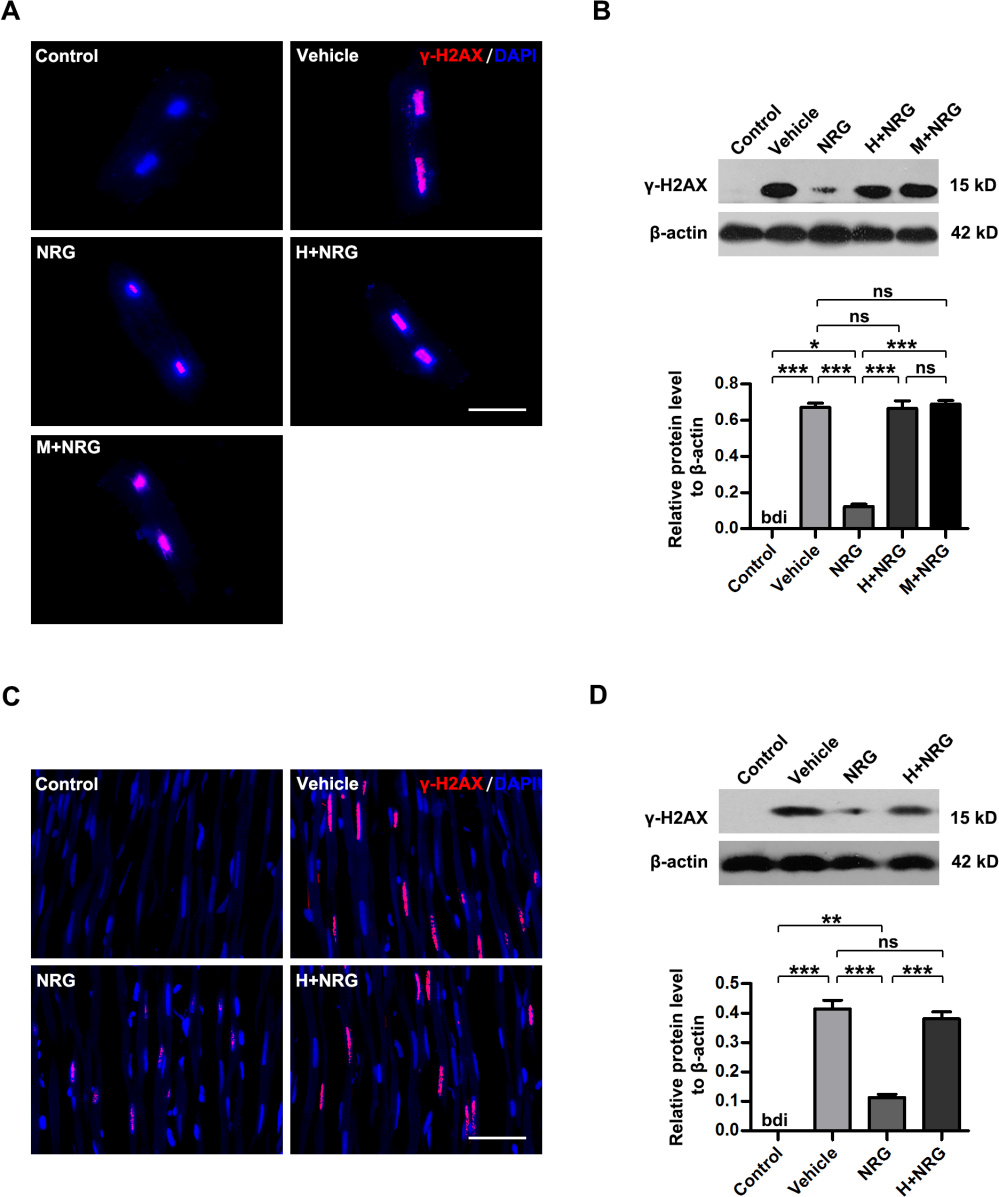
rhNRG-1β rescues radiation-induced myocardium injury via ErbB2 signaling. (A)&(C) Immunofluorescence images showing γH2AX (red) in the nuclei (blue) of cultured adult rat cardiomyocytes (A) and rat myocardial tissue (C). (B)&(D) Western blotting analyses of γH2AX levels in the adult rat cardiomyocyte cultures (B) and rat myocardial tissue (D). β-actin expression was analyzed as an internal control. For (B) and (D), data are expressed as mean ± SEM; statistical significance is determined by one-way ANOVA and the following Bonferroni’s multiple comparisons; bdi, below detectable limit; ***, P<0.001; n = 5 per group. Scale bar: (A) = 25 μm; (C) = 100 μm. Abbreviation: ANOVA, analysis of variance; bdi, below detectable limit; DAPI, 4′,6′-diamidino-2-phenylindole; ErbB2, human epidermal growth factor receptor-2; H+NRG, Herceptin plus recombinant human neuregulin; M+NRG, Mubritinib plus recombinant human neuregulin; NRG, recombinant human neuregulin; SEM, standard error of mean.

γH2AX is considered to be the specific marker of DNA double-strand break and an important indicator of radiation-induced cellular injury, the level of which is tightly associated cardiac function in injured heart [26, 27]. To investigate whether rhNRG-1β influences the cardiomyocyte injury caused by irradiation, irradiated cardiomyocytes were subjected to immunofluorescence staining for γH2AX 6 h after irradiation (Fig 1A, S1A Fig). In contrast to the blank control (Control), cultured cardiomyocytes subjected to a single 5 Gy dose of X-ray radiation showed obvious upregulation of nuclear γH2AX levels (Vehicle, P<0.001 versus Control; n = 5 per group). The fluorescence intensity of γH2AX in the nuclei of cardiomyocytes pretreated with rhNRG-1β was markedly reduced (NRG, P<0.001 versus Vehicle; n = 5 per group), which indicated attenuation of irradiation-induced damage. However, when cardiomyocytes were exposed to Herceptin (as a ErbB2 receptor antagonist) before rhNRG-1β treatment, the protection of cells against radiation-induced injury by rhNRG-1β was nearly abolished (H+NRG, P<0.001 versus NRG, P>0.05 versus Vehicle; n = 5 per group), indicating that rhNRG-1β functions via ErbB2. The irradiation-protective effects of rhNRG-1β was also abrogated by Mubritinib (a selective small molecule inhibitor of ErbB2), which rules out the specific effects of Herceptin (M+NRG, P<0.001 versus NRG, P>0.05 versus Vehicle, P>0.05 versus H+NRG; n = 5 per group). Consistent phenomena were observed in the myocardial tissue of irradiated rats (Fig 1C, S1B Fig). Immunohistochemical staining performed 6 h after radiation also showed that rhNRG-1β pretreatment ameliorated the myocardial injury caused by irradiation, but ErbB2 blockage by Herceptin nearly eliminated the protective effects of rhNRG-1β on the myocardium (Vehicle, P<0.001 versus Control; NRG, P<0.001 versus Vehicle; H+NRG, P<0.001 versus NRG, P>0.05 versus Vehicle; n = 5 per group). The rhNRG-1β/ErbB2-mediated protection of cultured adult cardiomyocyte and myocardium tissue against irradiation was further confirmed by Western blotting analyses for γH2AX (Fig 1B and 1D). Although γH2AX-indicative DNA damage is implicated in the cell necrosis and apoptosis in myocardium [27, 28], neither necrosis-related cell lysis and inflammatory response nor typical apoptotic body and apoptosis-specific activation of caspase 3 were detected in the irradiated cardiomyocyte cultures and rat myocardium tissue (Fig 1, S2 Fig). These results suggested that rhNRG-1β rescued radiation-induced injury in cultured cardiomyocytes and rat myocardial tissue, which was mediated by ErbB2 signaling.

### rhNRG-1β maintains mitochondrial homeostasis in irradiated cardiomyocytes

**Fig 2:**
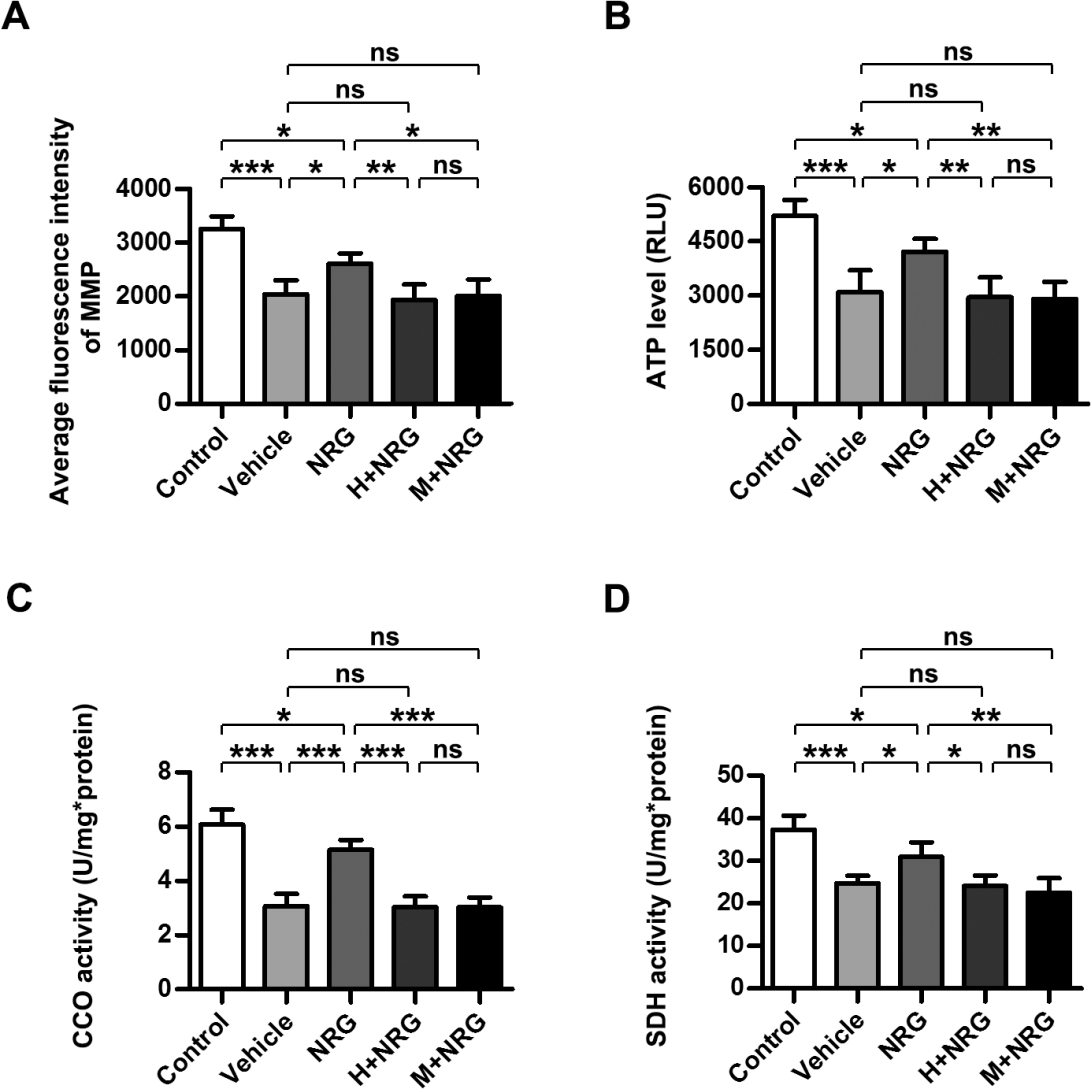
rhNRG-1β maintains mitochondrial homeostasis of irradiated cardiomyocyte. (A) Quantitative analysis of the fluorescence intensity of MMP (A), the ATP level (B), the CCO (C) and SDH activity (D) the in the adult rat cardiomyocyte cultures. Data are expressed as mean ± SD; statistical significance is determined by one-way ANOVA and the following Bonferroni’s multiple comparisons; ns, nonsignificant; *, P<0.05; **, P<0.01; ***, P<0.001; n = 5 per group. Abbreviation: ANOVA, analysis of variance; ATP, adenosine triphosphate; bdi, below detectable limit; CCO, cytochrome C oxidase; H+NRG, Herceptin plus recombinant human neuregulin; MMP, mitochondrial membrane potential; M+NRG, Mubritinib plus recombinant human neuregulin; NRG, recombinant human neuregulin; ns, nonsignificant; SD, standard deviation; SDH, succinic dehydrogenase.

The energy metabolism status and mitochondrial function of cardiomyocytes are important measurements of myocardial function, and mitochondrial dysfunction is implicated in a variety of myocardial injuries and cardiac diseases, including RIHD [29–31]. When mitochondrial dysfunction occurs (especially in the cardiomyocyte pathology), the mitochondrial membrane potential (MMP), which is generated with the electron transfer in the respiratory chain and serves as the driving force of ATP synthesis, is always disrupted [32, 33]. To examine whether rhNRG-1β could preserve mitochondrial homeostasis in irradiated cardiomyocytes, MMP of primary adult cardiomyocyte cultures was assayed 6 h after irradiation (Fig 2A). Consistent with the data from a previous study [29], the MMP of cultured cardiomyocytes subjected to 5 Gy radiation was obviously decreased (Vehicle; P<0.001 versus Control; n = 5 per group), whereas cells pretreated with rhNRG-1β showed significantly less reduction of the MMP, although some reduction was still observed (NRG, P<0.05 versus Vehicle, P<0.05 versus Control; n = 5 per group). However, the protection of the cardiomyocyte MMP by rhNRG-1β was abrogated by Herceptin or Mubritinib (H+NRG, P<0.01 versus NRG, P>0.05 versus Vehicle; M+NRG, P<0.05 versus NRG, P>0.05 versus Vehicle; n = 5 per group). ATP production, which is generally recognized as a direct reflection of mitochondrial function, was also measured 6 h after cardiomyocyte cultures were irradiated (Fig 2B). Similar to the results of the MMP assay, rhNRG-1β pre-treatment significantly attenuated the reduction of the cellular ATP level caused by 5 Gy radiation (Vehicle, P<0.001 versus Control; NRG, P<0.05 versus Vehicle, P<0.05 versus Control; n = 5 per group), but ErbB2 blockage through prior Herceptin or Mubritinib supplement vitiated the rescue of ATP production by rhNRG-1β (H+NRG, P<0.01 versus NRG, P>0.05 versus Vehicle; M+NRG, P<0.01 versus NRG, P>0.05 versus Vehicle; n = 5 per group).

The observations regarding MMP and ATP levels in cardiomyocyte cultures indicated that rhNRG-1β administration protected irradiated cardiomyocytes against the loss of mitochondrial homeostasis to a great degree, although not completely. To further confirm the protective effects of rhNRG-1β on the mitochondrial function and energy metabolism of cardiomyocytes receiving radiation, the activities of cytochrome *c* oxidase (CCO) and succinate dehydrogenase (SDH), which serve as indicators of the functional status of the respiratory chain and Krebs cycle, respectively [34, 35], were evaluated (Fig 2C and 2D). As expected, the impaired enzyme activities of CCO and SDH in irradiated cardiomyocytes were restored to approximately the 80% of normal levels by rhNRG-1β pre-treatment, but the therapeutic effects of rhNRG-1β were neutralized when Herceptin or Mubritinib treatment was applied in advance (For CCO activity, Vehicle, P<0.001 versus Control; NRG, P<0.001 versus Vehicle, P<0.05 versus Control; H+NRG, P<0.001 versus NRG, P>0.05 versus Vehicle; M+NRG, P<0.001 versus NRG, P>0.05 versus Vehicle; n = 5 per group. For SDH activity, Vehicle, P<0.001 versus Control; NRG, P<0.05 versus Vehicle, P<0.05 versus Control; H+NRG, P<0.05 versus NRG, P>0.05 versus Vehicle; M+NRG, P<0.01 versus NRG, P>0.05 versus Vehicle; n = 5 per group).

### rhNRG-1β preserves mitochondrial function in irradiated cardiomyocytes via the ERK-SIRT1 pathway

**Fig 3:**
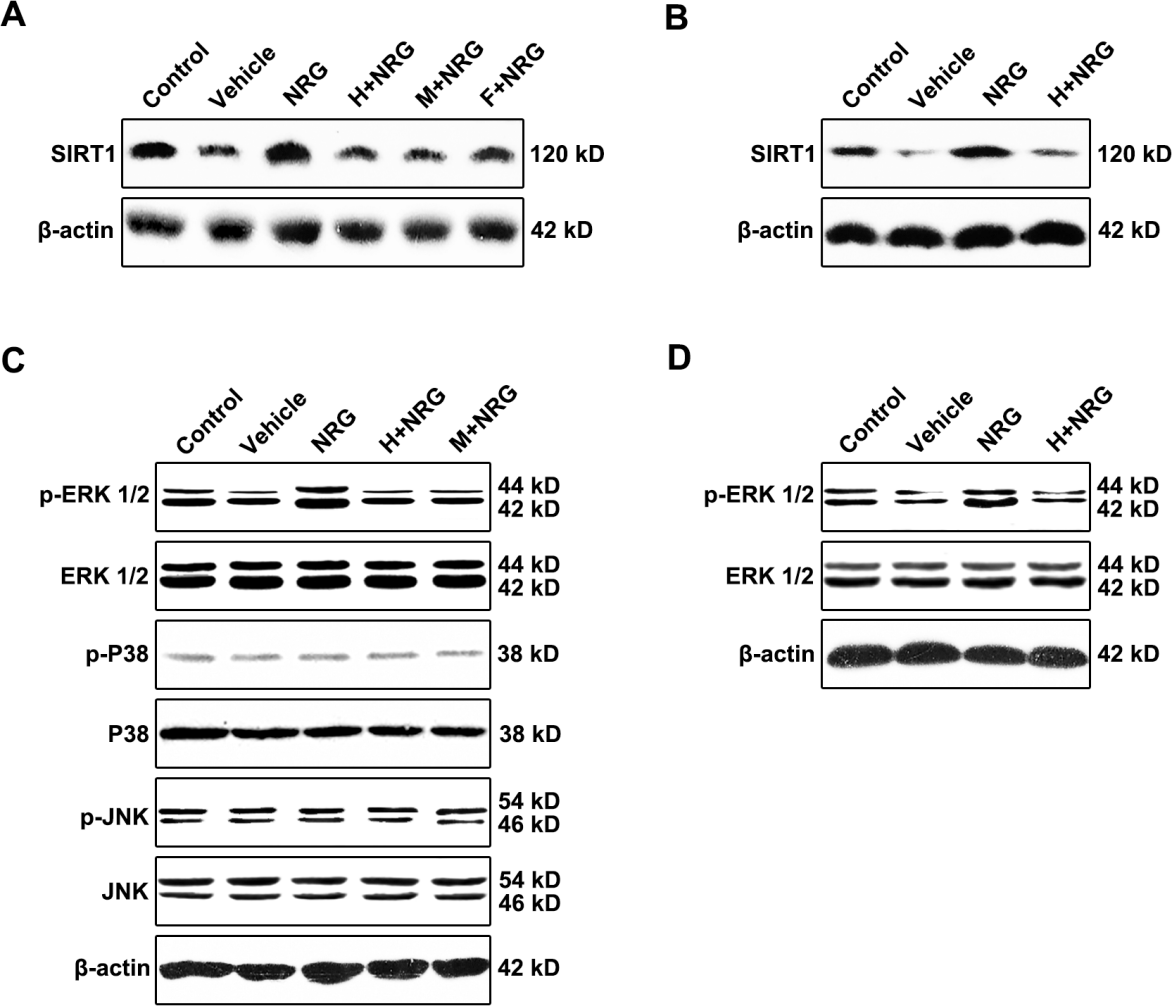
rhNRG-1β preserves mitochondrial function in irradiated cardiomyocyte via ERKs-SIRT1 pathway. (A-B) Western blots of SIRT1 in the adult rat cardiomyocyte cultures (A) and rat myocardial tissue (B). (C) Western analyses of the phosphorylation of ERK1/2, P38 and JNK in the adult rat cardiomyocyte cultures. (D) Western analyses of the phosphorylation of ERK1/2 in the rat myocardial tissue. β-actin expression was analyzed as an internal control. Abbreviation: F+NRG, FR180204 plus recombinant human neuregulin; H+NRG, Herceptin plus recombinant human neuregulin; M+NRG, Mubritinib plus recombinant human neuregulin; NRG, recombinant human neuregulin; ns, nonsignificant.

SIRT1, a ubiquitous NAD-dependent deacetylase, is considered to be an important positive regulator of mitochondrial integrity and homeostasis, the level of which is demonstrated to parallel mitochondrial function and energy metabolism status [36]. To explore the mechanism underlying the preservation of mitochondrial homeostasis in irradiated cardiomyocytes by rhNRG-1β, the expression of SIRT1 in cardiomyocyte cultures was assessed by Western blotting (Fig 3A, S3A Fig). Corresponding to the measurements of mitochondrial function, the suppressed expression of SIRT1 in cells subjected to radiation (Vehicle) was dramatically elevated due to pretreatment with rhNRG-1β, whereas the rhNRG-1β-induced upregulation of SIRT1 levels was almost abolished as a consequence of Herceptin or Mubritinib complementation, which was utilized for ErbB2 blockage (Vehicle, P<0.01 versus Control; NRG, P<0.001 versus Vehicle; H+NRG, P<0.001 versus NRG, P>0.05 versus Vehicle; M+NRG, P<0.001 versus NRG, P>0.05 versus Vehicle; n = 5 per group). The rhNRG-1β-triggered elevation of mitochondrial-protective factor SIRT1 expression which is mediated by ErbB2 signaling was also observed in the myocardium tissue of irradiated rats (Fig 3B, S3B Fig).

These findings suggested that the level of the mitochondrial protective factor SIRT1 in irradiated adult cardiomyocytes was significantly upregulated by NRG-1-activated ErbB2 signaling. Previous studies have shown that SIRT1 expression in cardiomyocytes and other cell lineages is positively modulated by MAPK (including ERKs, p38 and JNK) pathways, which specifically function as the downstream signaling molecules of NRG/ErbB2 [9, 37, 38]. Our data showed that in the cultured adult cardiomyocytes, the phosphorylation level of ERK1/2 was evidently reduced after irradiation, whereas rhNRG-1β treatment markedly upregulated the phosphorylation of ERK1/2, and as expected, the positive impact of rhNRG-1β was entirely abolished by prior application of Herceptin or Mubritinib (Vehicle, P<0.01 versus Control; NRG, P<0.001 versus Vehicle; H+NRG, P<0.001 versus NRG, P>0.05 versus Vehicle; M+NRG, P<0.001 versus NRG, P>0.05 versus Vehicle; n = 5 per group; Fig 3C, S3C Fig). The changes in the activation of ERK1/2 were highly consistent with the changes in SIRT1 expression in the cardiomyocyte cultures. However, no changes in the levels of activated p38 or JNK were detected under the current experimental conditions (For p38 and JNK activation: Vehicle, P>0.05 versus Control; NRG, P>0.05 versus Vehicle; H+NRG and M+NRG, P>0.05 versus NRG, P>0.05 versus Vehicle; n = 5 per group; Fig 3C, S3D and S3E Fig). Consistently, the NRG/ErbB2 signaling-activated phosphorylation of ERK1/2 was also detected in the irradiated rat-derived myocardium tissue (Fig 3D, S3F Fig). To further examine whether the rhNRG-1β-induced elevation of SIRT1 levels in cardiomyocytes subjected to radiation was mediated by ERK signaling, a specific antagonist of ERKs, FR180204, was added to the cardiomyocyte culture before rhNRG-1β supplementation. Western blot data showed that inhibition of ERK activation with FR180204 (F+NRG) was sufficient to abolish the augmented expression of SIRT1 caused by rhNRG-1β (F+NRG, P<0.001 versus NRG, P>0.05 versus Vehicle; n = 5 per group; Fig 3A, S3A Fig). These results indicated that the protection of mitochondrial homeostasis and energy metabolism by rhNRG-1β in irradiated cardiomyocytes and myocardium tissue was mediated, at least in part, by the ERK-SIRT1 pathway.

### rhNRG-1β exerts long-term protective effects on the irradiated myocardium

**Fig 4:**
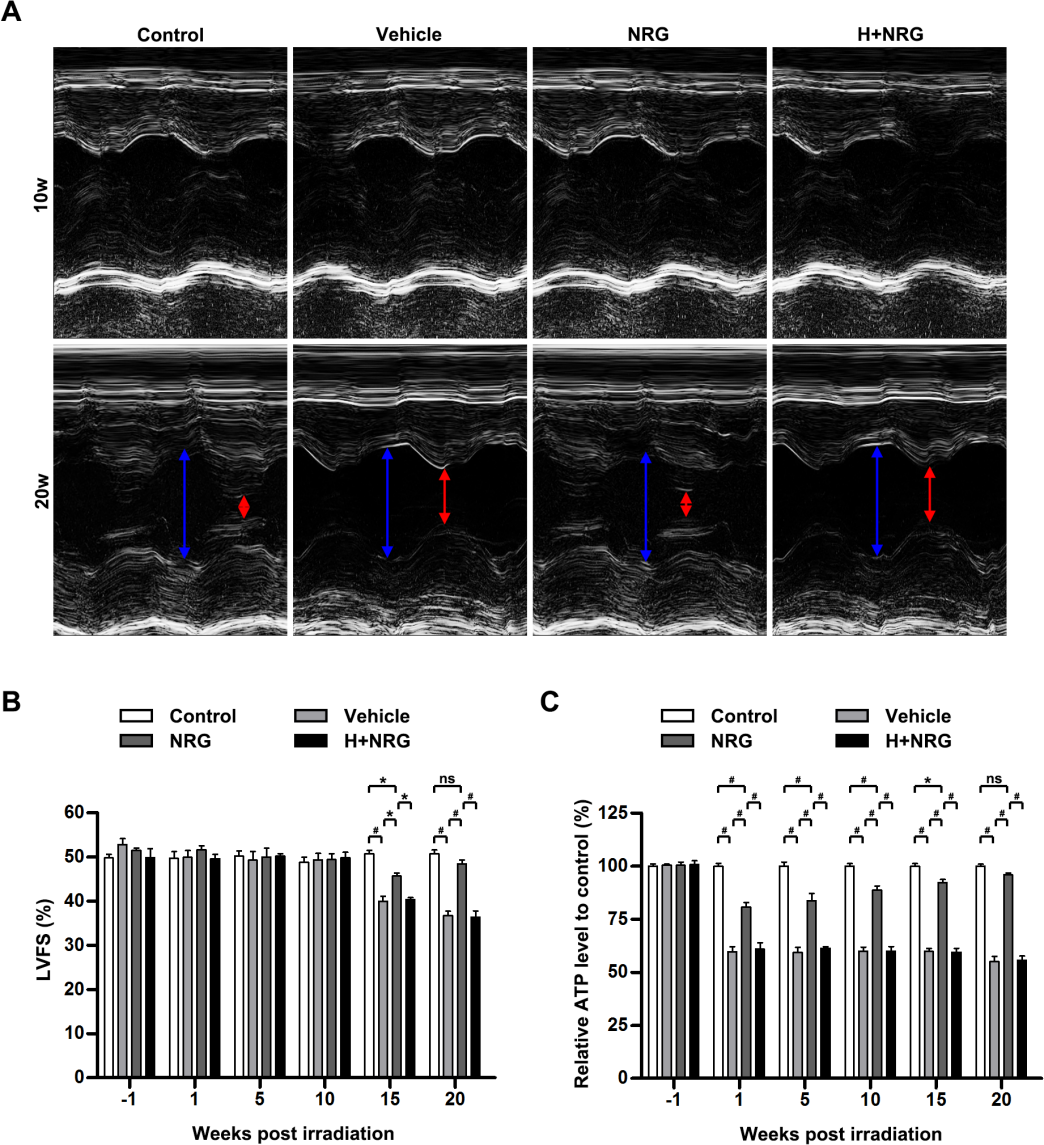
rhNRG-1β exerts long-term protective effects on the function of irradiated myocardium. (A) Representative M-mode images of left ventricles of the irradiated rats at Weeks 10 and 20 post-irradiation. (B) Quantitative analysis of LVFS of the irradiated rats at Week 1 pre-irradiation and Weeks 1, 5, 10, 15 and 20 post-irradiation. (C) Quantitative analysis of the relative ATP level in myocardial tissue of the irradiated rats at Week 1 pre-irradiation and Weeks 1, 5, 10, 15 and 20 post-irradiation. For (B) and (C), data are expressed as mean ± SEM; statistical significance is determined by two-way ANOVA and the following Bonferroni’s multiple comparisons; ns, nonsignificant; *, P<0.05; #, P<0.001; n = 4 per group per time point. Abbreviation: ANOVA, analysis of variance; ATP, adenosine triphosphate; H+NRG, Herceptin plus recombinant human neuregulin; LVFS, left ventricular fractional shortening; M+NRG, Mubritinib plus recombinant human neuregulin; NRG, recombinant human neuregulin; ns, nonsignificant; SEM, standard error of the mean.

To investigate the protective effects of rhNRG-1β on the cardiac function of rats that received a single 20 Gy dose of radiation, the pump function of the left ventricle (LV) was monitored during the entire observation period via echocardiography and presented in M-Mode echocardiographic images at the mid-papillary muscle level of the LV. As shown in Fig 4A and 4B, LV fractional shortening (FS) remained similar in the Control group, the Vehicle group, the NRG group and the H+NRG group until 10 weeks post-irradiation. However, at the 15-week time point, animals in the Vehicle group that received only radiation without any treatment exhibited a decline in LVFS (Control: 50.78±1.49%; Vehicle: 40.01±2.28%; Vehicle, P<0.001 versus Control; n = 4 per group). The reduction of LVFS was significantly attenuated by rhNRG-1β medication (NRG: 50.78±1.49%; NRG, P<0.05 versus Vehicle; n = 4 per group), whereas the effect was totally neutralized using Herceptin (H+NRG: 40.38±0.93%; H+NRG, P<0.05 versus NRG; n = 4 per group). On the 20th week post-irradiation, the animals in the Vehicle group and the H+NRG group still displayed impaired LV pump function (Control: 50.83±1.64%; Vehicle: 36.72±2.03%; H+NRG: 36.40±2.76%; Vehicle, P<0.001 versus Control; n = 4 per group). In contrast, the rats in the NRG group, which received short-term rhNRG-1β treatment in the peri-radiation period, regained the normal level of LVFS (NRG: 48.47±1.61%; NRG, P<0.001 versus Vehicle; H+NRG, P<0.001 versus NRG; n = 4 per group). The long-term protective effects of NRG/ErbB2 signaling on cardiac function of irradiated rats were also revealed by the measurement of other indicators of blood-pumping function, left ventricular end-systolic volume (LVESV; S4A Fig) and LV ejection fraction (LVEF; S4C Fig), though monitoring of left ventricular end-diastolic volume (LVEDV) determined no between-group difference at each time point (S4B Fig).

Distinct from the phenomena observed regarding cardiac pump function, the observed alterations of myocardial ATP production occurred in the rats immediately after irradiation (Fig 4C). Compared with the rats in the Control group, animals in the Vehicle group exhibited dramatically reduced myocardial ATP levels throughout the observation period Vehicle, P<0.001 versus Control at 1, 5, 10, 15 and 20 weeks following radiation; n = 4 per group per time point), whereas continuous recovery of ATP production occurred in the myocardium tissue of rhNRG-1β-treated animals until 20 weeks post-radiation, when myocardial ATP synthesis was restored to the physiological level (NRG, P<0.001 versus Vehicle at 1, 5, 10, 15 and 20 weeks following radiation, P<0.001 versus Control at 1, 5 and 10 weeks following radiation, P<0.05 versus Control at 15 weeks following radiation, P>0.05 versus Control on 20 weeks following radiation; n = 4 per group per time point). Concurring with expectations, the restoration of ATP levels in the irradiated myocardium of rhNRG-1β-treated rats was completely ablated by the prior injection of Herceptin (H+NRG, P<0.001 versus NRG at 1, 5, 10, 15 and 20 weeks following radiation; n = 4 per group per time point). These data suggested that rhNRG-1β treatment could protect the irradiation-induced abnormalities of cardiac pump function and the myocardial energy metabolism over a long period of time.

**Fig 5:**
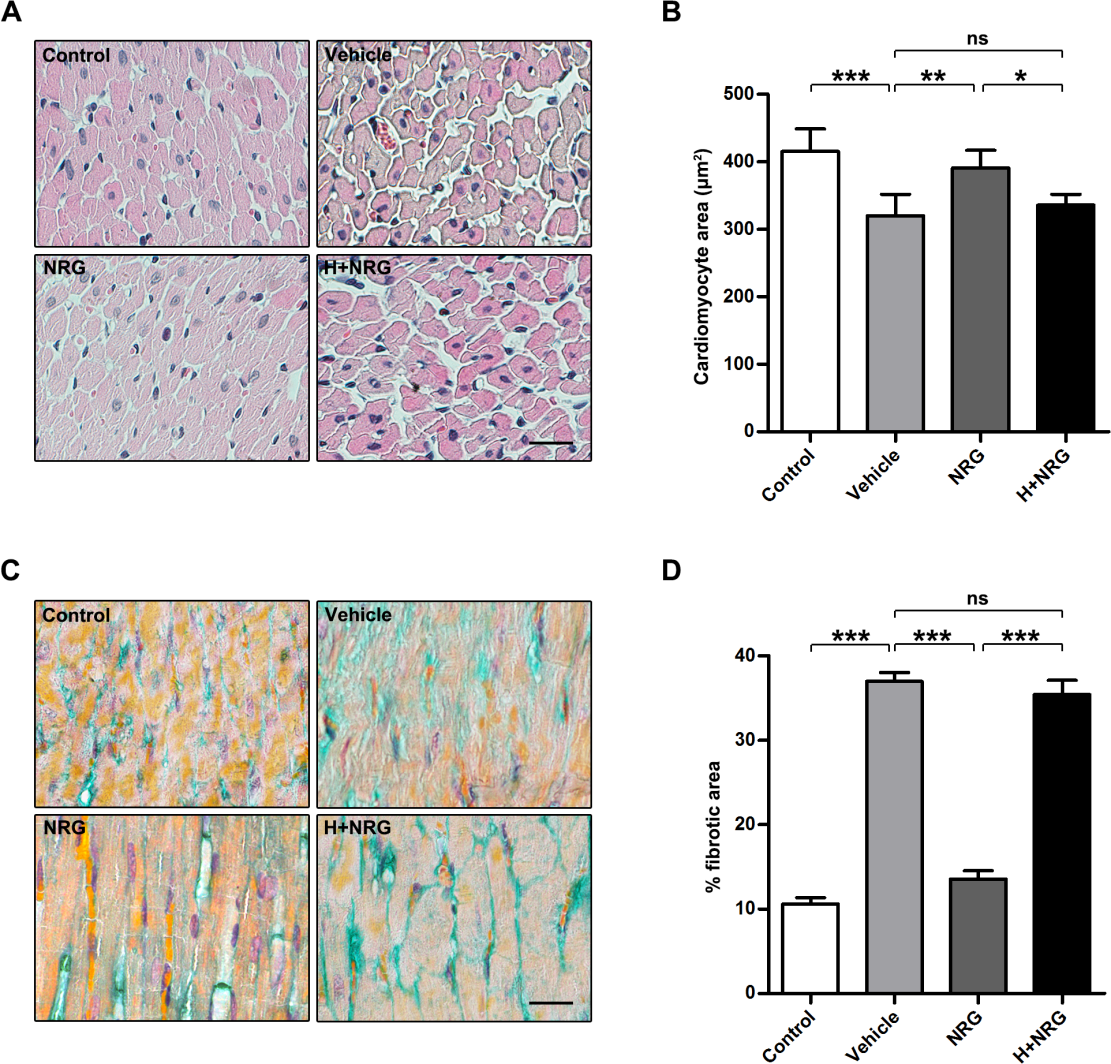
rhNRG-1β exerts long-term protective effects on the morphology of irradiated myocardium. (A) Images of HE-stained sections of rat myocardial tissue dissected 20 weeks after irradiation. (B) Quantification of the cardiomyoctye area in the sections of rat myocardial tissue dissected 20 weeks after irradiation. (C) Images of Masson-stained sections of rat myocardial tissue dissected 20 weeks after irradiation. Fibrosis is determined by light green staining. (D) Quantification of the fibrosis in the sections of rat myocardial tissue dissected 20 weeks after irradiation. For (B), data are expressed as mean ± SD; For (D), data are expressed as mean ± SEM; statistical significance is determined by one-way ANOVA and the following Bonferroni’s multiple comparisons; *, P<0.05; **, P<0.01; ***, P<0.001; n = 4 per group. Scale bar: (A) and (B) = 25 μm. Abbreviation: ANOVA, analysis of variance; H+NRG, Herceptin plus recombinant human neuregulin; M+NRG, Mubritinib plus recombinant human neuregulin; HE, hematoxylin-eosin; NRG, recombinant human neuregulin; ns, nonsignificant; SD, standard deviation; SEM, standard error of the mean.

Previous studies have found that irradiation-induced deficiency of cardiac function detected in the late stage of RIHD is always accompanied by general cardiomyocyte hypotrophy and concomitant diffused myocardial fibrosis [1]. To assess whether rhNRG-1β treatment could ameliorate the long-term deterioration of myocardial structure, the remaining rats in each group were euthanized at the end of the observation period, and their dissected hearts were subjected to histological assays. In HE-stained sections (Fig 5A and 5B), the transverse cross-sectional area of cardiomyocytes was evidently reduced 20 weeks after the initial 20 Gy irradiation (Control: 415.1±33.24 μm^2^; Vehicle: 319.6±32.11 μm^2^; Vehicle, P<0.001 versus Control; n = 4 per group). Irradiation-induced cardiomyocyte hypotrophy was significantly attenuated by rhNRG-1β treatment applied around the irradiation (NRG: 390.6±11.7 μm^2^; NRG, P<0.01 versus Vehicle; n = 4 per group), but rhNRG-1β failed to protect the irradiated myocardium from hypotrophy when ErbB2 was blocked by Herceptin in advance (H+NRG: 335.4±16.37 μm^2^; H+NRG, P<0.05 versus NRG, P>0.05 versus Vehicle; n = 4 per group). Interstitial fibrosis was examined via Masson’s trichrome staining (Fig 5C and 5D). Our data showed that severe fibrosis (determined by light green staining) in the irradiated myocardium did not occur in the myocardium tissue of the rats receiving rhNRG-1β treatment (for the percentage of fibrotic area, Control: 10.58±0.77%; Vehicle: 36.95±1.06%; NRG: 13.56±0.98%; Vehicle, P<0.001 versus Control; NRG, P<0.001 versus Vehicle; n = 4 per group). As expected, the beneficial effects of rhNRG-1β were undetectable when Herceptin was administered prior to rhNRG-1β treatment (H+NRG: 35.41±1.69%; H+NRG, P<0.001 versus NRG, P>0.05 versus Vehicle; n = 4 per group). Hence, the long-term protective effects of rhNRG-1β on the structure of the irradiated myocardium were demonstrated by the data obtained through HE and Masson’s trichrome staining, which are consistent with and confirmed by each other.

## Discussion

RIHD is a serious side effect of the radiotherapy applied for the treatment of various tumors, during which the heart is inevitably irradiated [1, 2]. However, other than reducing the radiation dose and field at the expense of decreased therapeutic efficacy, current clinical methods remain ineffective in preventing and treating RIHD. Hence, it is necessary to develop new therapies. Recent studies show that rhNRG-1β can improve cardiac function and survival in animal models of ischemic heart disease, doxorubicin-induced heart damage and heart failure [7, 9, 12–14]. In particular, rhNRG-1β has been confirmed to improve cardiac function in chronic heart failure patients [15]. In the present study, we first demonstrate that rhNRG-1β effectively protects the myocardium against irradiation-induced damage, maintains cardiomyocyte mitochondrial homeostasis and preserves cardiac pump function. Moreover, the protection of the irradiated myocardium by rhNRG-1β depends on the activation of the ErbB2-ERK-SIRT1 signaling pathway.

Studies on the treatment of ischemic heart disease and heart failure using rhNRG-1β indicate that rhNRG-1β can improve the survival and contractility of cardiomyocytes [7, 9, 14]. Additionally, rhNRG-1β has been shown to promote cardiomyocyte dedifferentiation and proliferation in adult rat hearts via the NRG/ErbB2 signaling pathway [9, 38]. NRG1 stimulates karyokinesis, DNA synthesis and cytokinesis in cultured adult rat ventricular cardiomyocytes [7]. However, increases in reactive oxygen species, oxidative DNA damage, and DNA damage responses shorten the postnatal proliferative window of cardiomyocytes [39]. The present study shows that rhNRG-1β significantly ameliorates the myocardial nuclear DNA damage caused by irradiation both *in vitro* and *in vivo*. Therefore, we infer that rhNRG-1β may facilitate the repair of DNA damage but does not open the proliferative window of cardiomyocytes after irradiation. Interestingly, no distinct necrosis and apoptosis of cardiomyocytes (Fig 1, S2 Fig) or change in echocardiography was observed in the early stage (Fig 4, S4 Fig). These results suggest that the myocardial nuclear DNA lesions represent subclinical damage, which may exert long-time impacts on the metabolism and function of cardiomyocytes.

Local heart irradiation leads to mitochondrial damage, respiratory chain impairment and increases in oxidized proteins [29, 40]. Thus, the present study was focused on the protective effects of rhNRG-1β on cardiomyocyte mitochondrial homeostasis during irradiation. Previous studies have shown that SIRT1, a ubiquitous NAD-dependent deacetylase, which is distributed in both the nucleus and cytoplasm and is involved in longevity and stress responses [41, 42], also plays a critical role in the maintenance of mitochondrial integrity and metabolism in adult cardiomyocytes [43]. Activating SIRT1 restrains the irradiation-induced inflammatory reaction in mesenchymal stem cells and antagonizes oxidation in the hippocampus resulting from radiation [44]. Our findings show that rhNRG-1β effectively ameliorates the impairment of mitochondrial functions following irradiation, which is coupled with upregulated expression of SIRT1, suggesting the involvement of SIRT1 in mitochondrial protection by rhNRG-1β. As revealed in previous research, the expression of SIRT1 is significantly modulated by MAPK pathways (especially ERK/MEK pathway), which also mediate the intracellular transduction of the NRG/ErbB2 proliferative signal in cardiomyocytes. Our results demonstrate that in the protection of the irradiated myocardium, the rhNRG-1β-induced elevation of SIRT1 levels is mediated, at least in part, via the activation of ERKs (Fig 3, S3 Fig). Interestingly, we did not detect obvious changes in the P38 and JNK signaling pathways (Fig 3, S3 Fig), which may suggest the particularity of the regulation of SIRT1 expression in RIHD. In addition, SIRT1 and Tip60 cooperatively regulate DDR signaling and DNA repair by controlling the state of H2AX acetylation [45]. Hence, we propose that the alleviation of nuclear lesions in irradiated cardiomyocytes pre-treated with rhNRG–1β results from accelerated DNA damage repair, which is also mediated by the rhNRG–1β-activated ERK-SIRT1 signaling pathway.

Subnormal left ventricle function following irradiation is observed after many years in breast cancer patients [46]. Consistent with clinical observations, we found that the alteration of left ventricle functionality was significantly delayed, in contrast to the decline in myocardium ATP levels during the development of RHID. Sridharan *et al* showed that long-term changes in mitochondrial membrane functions, the oxygen consumption rate and SDH levels take place following heart irradiation [29]. We infer that the subclinical lesions of nuclear DNA and mitochondria in irradiated myocardia gradually accumulate and ultimately result in the impairment of cardiac function and structural deterioration (such as myocardium degeneration and myocardial fibrosis; Fig 5). It is encouraging to find that with rhNRG–1β administration, the ATP level in the irradiated myocardium was markedly increased throughout the entire observation period, gradually being restored to normal levels, coupled with significantly improved left-ventricle functionality at a late stage (Fig 4, S4 Fig). These results demonstrate that rhNRG–1β partly prevented the deleterious cardiac effects of irradiation and exerted a long-term protective effect on the myocardium in RHID. These findings are in accord with the results of clinical studies on chronic heart failure, which show that short-term administration of rhNRG–1β is sufficient to improve cardiac function and to alleviate ventricular remodeling [15]. It has been demonstrated by this and other studies [7, 9, 12–15] that rhNRG–1β is a promising candidate for the treatment of RHID.

Because rhNRG-1β shows promise regarding the treatment of RHID, its potential to stimulate tumor growth must be taken into consideration. Although some studies have proposed that NRG1 may promote tumor growth, NRG1 has also been demonstrated to act as a principal tumor suppressor in many cancers [47, 48]. We did not observe any evidence of accelerated tumor growth caused by rhNRG–1β in breast cancer xenograft tumors in mice (data not shown). Further studies will need to focus on the effects of rhNRG-1β on other types of tumor cell lines.

In most studies addressing the impacts of NRG-1 on the myocardium, neonatal rat or mouse-derived cardiomyocytes have been selected as the *in vitro* model. Although the tyrosine kinase receptors of NRG-1 (including ErbB2, ErbB3 and ErbB4) are all implicated in the early heart development, NRG-1 only functions via ErbB2 and ErbB4, rather than ErbB3 in mature adult ventricular myocytes [49], which suggests that the examination of neonatal cardiomyocytes may not accurately reflect the effects of NRG-1 on the myocardium of an adult receiving radiotherapy. To overcome this obstacle, in the present study, cardiomyocytes isolated from adult rats were used as the cell model to assay the protective effects of rhNRG-1β. Moreover, in terms of animal irradiation models, the total body, or lower hemi-body, irradiation applied in previous studies frequently induces pulmonary hypertension and right-ventricle hypertrophy in an irradiation dose-dependent manner, which may make experimental observations and data analysis difficult [50] or lead to kidney lesions that increase the mortality of experimental animals [51]. In contrast, in the present study, the radiation field was minimized through accurate localization to reduce the radiation damage to other organs and control the experimental variants as far as possible.

## Conclusions

In summary, rhNRG-1β effectively ameliorates irradiation-induced myocardial nuclear damage, maintains mitochondrial homeostasis in irradiated cardiomyocytes and exerts long-term protective effects on the myocardial energy metabolism, cardiac pump function and the structure of myocardium tissue via NRG/ErbB2 signaling. Additionally, the protection of irradiated myocardium by rhNRG-1β is mediated, at least in part, by the activation of ErbB2-ERK-SIRT1 signaling transduction.

## Supporting Information

S1 Fig Quantification of the fluorescence intensity of γH2AX in Fig 1. Quantification of the fluorescence intensity of γH2AX in the adult rat cardiomyocyte cultures (A) and rat myocardial tissue (B). Data are expressed as mean ± SD; statistical significance is determined by one-way ANOVA and the following Bonferroni’s multiple comparisons; bdi, below detectable limit; ***, P<0.001; n = 5 per group. Abbreviation: ANOVA, analysis of variance; bdi, below detectable limit; DAPI, 4′,6′-diamidino-2-phenylindole; ErbB2, human epidermal growth factor receptor-2; H+NRG, Herceptin plus recombinant human neuregulin; M+NRG, Mubritinib plus recombinant human neuregulin; NRG, recombinant human neuregulin; SEM, standard error of mean.
(TIF)

S2 Fig Western blotting analyses of the activation of caspase 3. Western blotting analyses of cleaved caspase 3 and pro-caspase 3 in the adult rat cardiomyocyte cultures (B) and the rat myocardial tissue (D). Doxorubicin-treated cells and animals are used as the positive controls. β-actin expression was analyzed as an internal control. Abbreviation: DOX, Doxorubicin; H+NRG, Herceptin plus recombinant human neuregulin; M+NRG, Mubritinib plus recombinant human neuregulin; NRG, recombinant human neuregulin.
(TIF)

S3 Fig Quantification of the relative protein expression in Fig 3. Quantification of SIRT1 expression in the adult rat cardiomyocyte cultures (A) and the rat myocardial tissue (B). (C-E) Quantification of the ratios of the phosphorylated protein level to total protein level of ERK1/2, P38 and JNK in the adult rat cardiomyocyte cultures. (F) Quantification of the ratios of the phosphorylated protein level to total protein level of ERK1/2 in the the rat myocardial tissue. Data are expressed as mean ± SEM; statistical significance is determined by one-way ANOVA and the following Bonferroni’s multiple comparisons; ns, nonsignificant; **, p < 0.01; ***, p < 0.001; n = 5 per group. Abbreviation: ANOVA, analysis of variance; F+NRG, FR180204 plus recombinant human neuregulin; H+NRG, Herceptin plus recombinant human neuregulin; M+NRG, Mubritinib plus recombinant human neuregulin; NRG, recombinant human neuregulin; Saem, standard error of mean.
(TIF)

S4 Fig Quantitative analysis of LVESV, LVEDV and ejection fraction of the irradiated rats. Quantitative analysis of LVESV (A), LVEDV (B) and LVEF (C) of the irradiated rats at Week 1 pre-irradiation and Weeks 1, 5, 10, 15 and 20 post-irradiation. Data are expressed as mean ± SEM; statistical significance is determined by two-way ANOVA and the following Bonferroni’s multiple comparisons; ns, nonsignificant; *, P<0.05; **, P<0.01; #, P<0.001; n = 4 per group per time point. Abbreviation: ANOVA, analysis of variance; H+NRG, Herceptin plus recombinant human neuregulin; LVEDV, left ventricular end-diastolic volume; LVEF, left ventricular ejection fraction; LVESV, left ventricular end-systolic volume; M+NRG, Mubritinib plus recombinant human neuregulin; NRG, recombinant human neuregulin; SEM, standard error of the mean.
(TIF)
