# Supplementary figures and images for: RhNRG-1β Protects the Myocardium against Irradiation-Induced Damage via the ErbB2-ERK-SIRT1 Signaling Pathway
Source: PLoS One. 2015 Sep 2;10(9):e0137337. doi: 10.1371/journal.pone.0137337 (PMC4558028; doi:10.1371/journal.pone.0137337)

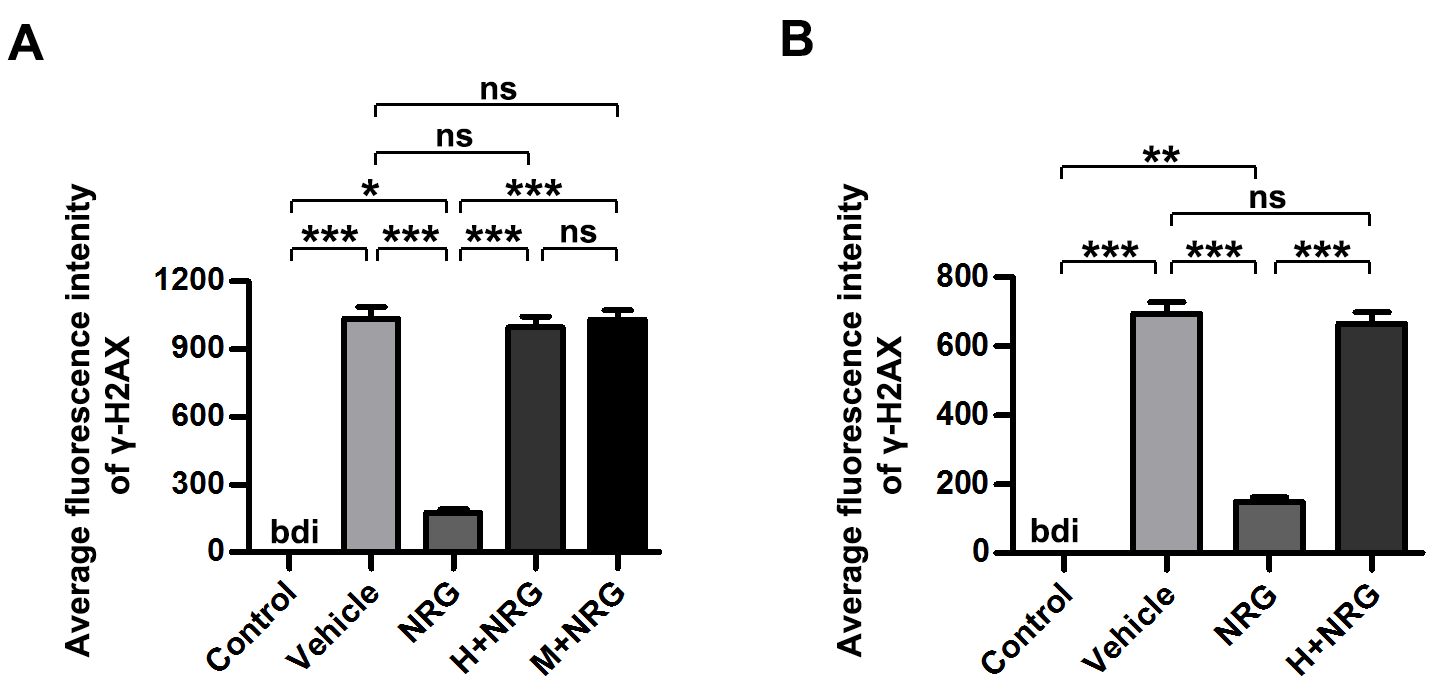

Supplement: S1 Fig — Quantification of the fluorescence intensity of γH2AX in the adult rat cardiomyocyte cultures (A) and rat myocardial tissue (B). Data are expressed as mean ± SD; statistical significance is determined by one-way ANOVA and the following Bonferroni’s multiple comparisons; bdi, below detectable limit; ***, P<0.001; n = 5 per group. Abbreviation: ANOVA, analysis of variance; bdi, below detectable limit; DAPI, 4′,6′-diamidino-2-phenylindole; ErbB2, human epidermal growth factor receptor-2; H+NRG, Herceptin plus recombinant human neuregulin; M+NRG, Mubritinib plus recombinant human neuregulin; NRG, recombinant human neuregulin; SEM, standard error of mean. (TIF) [file pone.0137337.s001.tif]

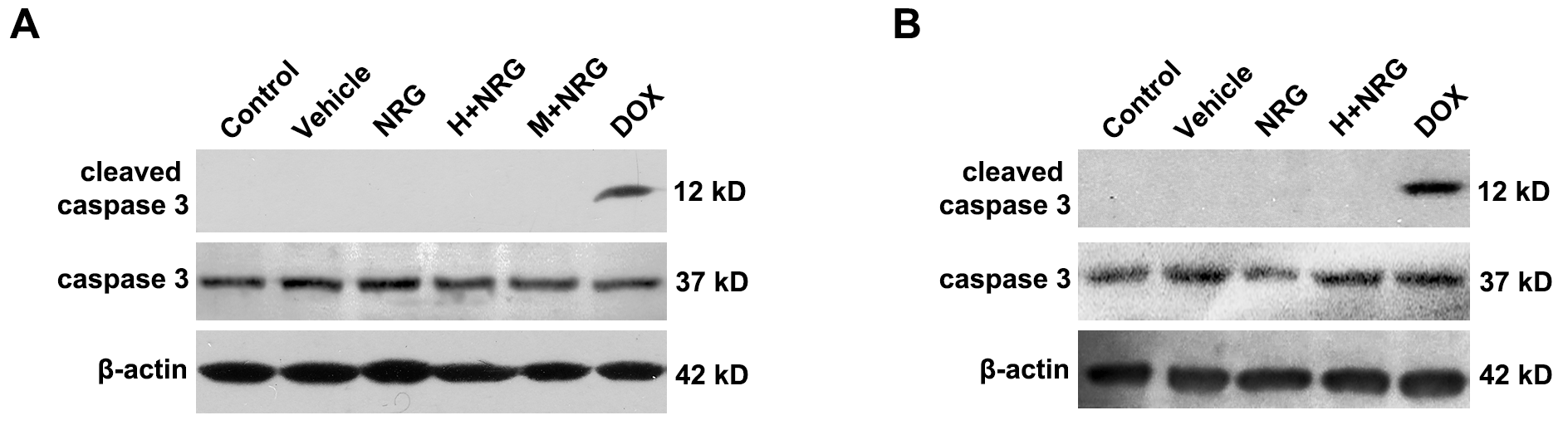

Supplement: S2 Fig — Western blotting analyses of cleaved caspase 3 and pro-caspase 3 in the adult rat cardiomyocyte cultures (B) and the rat myocardial tissue (D). Doxorubicin-treated cells and animals are used as the positive controls. β-actin expression was analyzed as an internal control. Abbreviation: DOX, Doxorubicin; H+NRG, Herceptin plus recombinant human neuregulin; M+NRG, Mubritinib plus recombinant human neuregulin; NRG, recombinant human neuregulin. (TIF) [file pone.0137337.s002.tif]

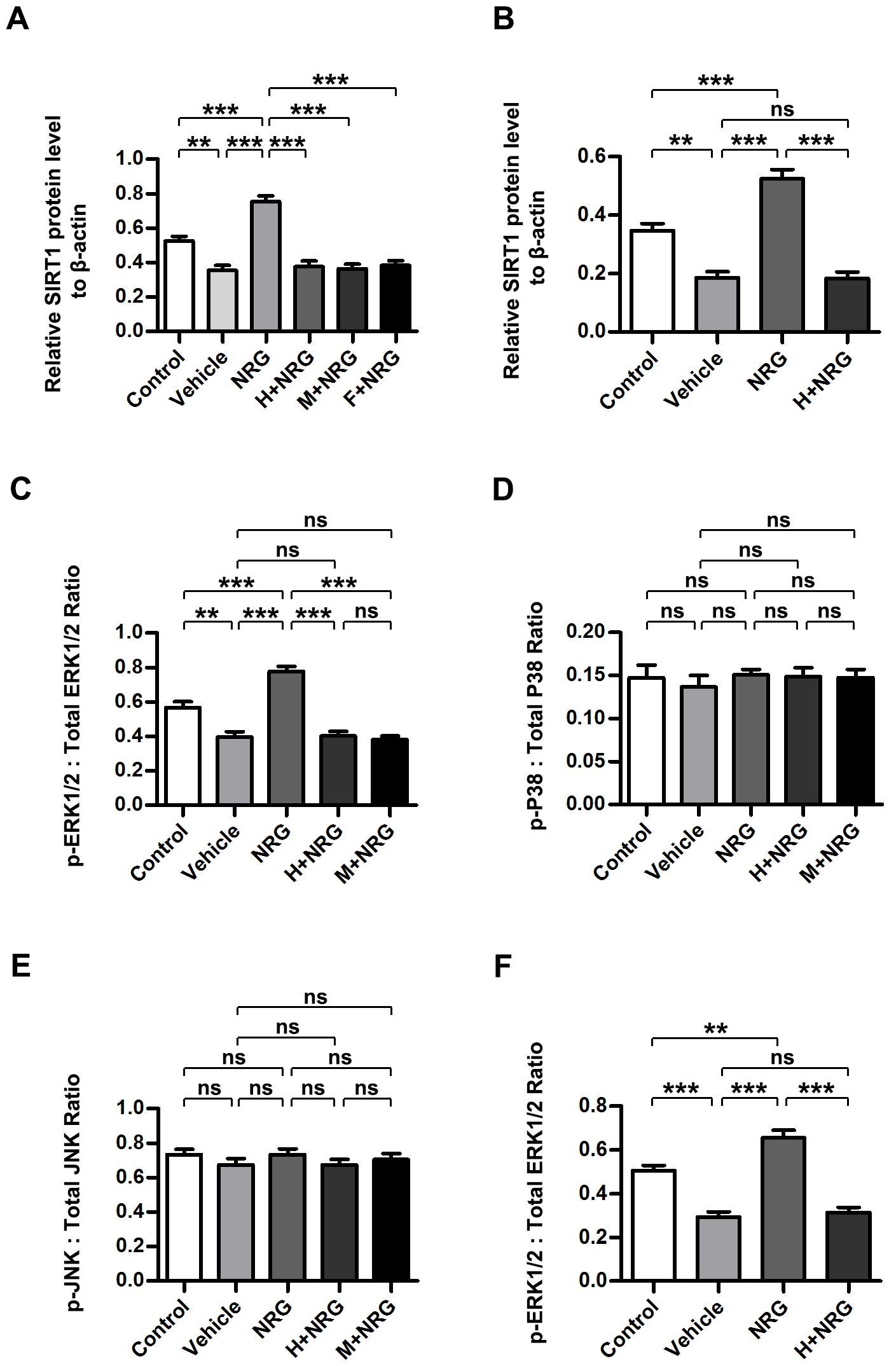

Supplement: S3 Fig — Quantification of SIRT1 expression in the adult rat cardiomyocyte cultures (A) and the rat myocardial tissue (B). (C-E) Quantification of the ratios of the phosphorylated protein level to total protein level of ERK1/2, P38 and JNK in the adult rat cardiomyocyte cultures. (F) Quantification of the ratios of the phosphorylated protein level to total protein level of ERK1/2 in the the rat myocardial tissue. Data are expressed as mean ± SEM; statistical significance is determined by one-way ANOVA and the following Bonferroni’s multiple comparisons; ns, nonsignificant; **, p < 0.01; ***, p < 0.001; n = 5 per group. Abbreviation: ANOVA, analysis of variance; F+NRG, FR180204 plus recombinant human neuregulin; H+NRG, Herceptin plus recombinant human neuregulin; M+NRG, Mubritinib plus recombinant human neuregulin; NRG, recombinant human neuregulin; Saem, standard error of mean. (TIF) [file pone.0137337.s003.tif]

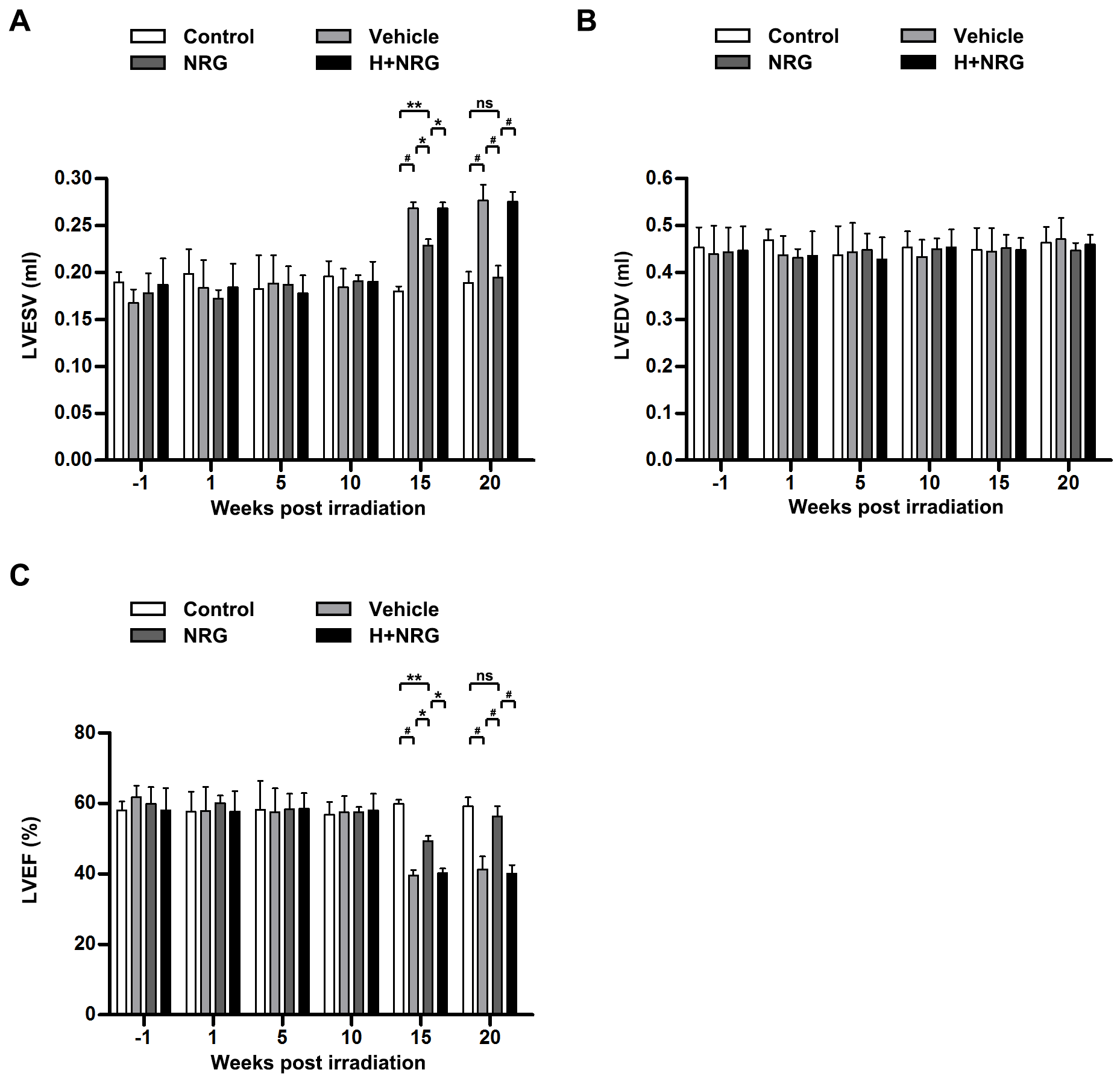

Supplement: S4 Fig — Quantitative analysis of LVESV (A), LVEDV (B) and LVEF (C) of the irradiated rats at Week 1 pre-irradiation and Weeks 1, 5, 10, 15 and 20 post-irradiation. Data are expressed as mean ± SEM; statistical significance is determined by two-way ANOVA and the following Bonferroni’s multiple comparisons; ns, nonsignificant; *, P<0.05; **, P<0.01; #, P<0.001; n = 4 per group per time point. Abbreviation: ANOVA, analysis of variance; H+NRG, Herceptin plus recombinant human neuregulin; LVEDV, left ventricular end-diastolic volume; LVEF, left ventricular ejection fraction; LVESV, left ventricular end-systolic volume; M+NRG, Mubritinib plus recombinant human neuregulin; NRG, recombinant human neuregulin; SEM, standard error of the mean. (TIF) [file pone.0137337.s004.tif]
